# Perifosine and CCI 779 Co-Operate to Induce Cell Death and Decrease Proliferation in PTEN-Intact and PTEN-Deficient PDGF-Driven Murine Glioblastoma

**DOI:** 10.1371/journal.pone.0014545

**Published:** 2011-01-18

**Authors:** Kenneth L. Pitter, Craig J. Galbán, Stefanie Galbán, Omid Saeed-Tehrani, Fei Li, Nikki Charles, Michelle S. Bradbury, Oren J. Becher, Thomas L. Chenevert, Alnawaz Rehemtulla, Brian D. Ross, Eric C. Holland, Dolores Hambardzumyan

**Affiliations:** 1 Department of Cancer Biology and Genetics, Memorial Sloan-Kettering Cancer Center, New York, New York, United States of America; 2 Brain Tumor Center, Memorial Sloan-Kettering Cancer Center, New York, New York, United States of America; 3 Departments of Radiology, The University of Michigan Medical School, Ann Arbor, Michigan, United States of America; 4 Radiation Oncology, The University of Michigan Medical School, Ann Arbor, Michigan, United States of America; 5 Department of Radiology, Memorial Sloan-Kettering Cancer Center, New York, New York, United States of America; 6 Department of Pediatrics, Duke University Medical Center, Durham, North Carolina, United States of America; 7 Preston Robert Tisch Brain Tumor Center, Duke University Medical Center, Durham, North Carolina, United States of America; 8 Departments of Neurosurgery, Neurology and Surgery, Memorial Sloan-Kettering Cancer Center, New York, New York, United States of America; 9 Department of Stem Cell Biology and Regenerative Medicine in Lerner Research Institute at Cleveland Clinic, Cleveland, Ohio, United States of America; Roswell Park Cancer Institute, United States of America

## Abstract

**Background:**

Platelet derived growth factor receptor (PDGFR) activity is deregulated in human GBM due to amplification and rearrangement of the PDGFR-alpha gene locus or overexpression of the PDGF ligand, resulting in the activation of downstream kinases such as phosphatidylinositol 3-kinase (PI3K), Akt, and mammalian target of rapamycin (mTOR). Aberrant PDGFR signaling is observed in approximately 25-30% of human GBMs, which are frequently molecularly classified as the proneural subclass. It would be valuable to understand how PDGFR driven GBMs respond to Akt and mTOR inhibition.

**Methodology/Principal Findings:**

Using genetically engineered PTEN-intact and PTEN-deficient PDGF-driven mouse models of GBM that closely mimic the histology and genetics of the human PDGF subgroup, we investigated the effect of inhibiting Akt and mTOR alone or in combination *in vitro* and *in vivo*. We used perifosine and CCI-779 to inhibit Akt and mTOR, respectively. Here, we show *in vitro* data demonstrating that the most effective inhibition of Akt and mTOR activity in both PTEN-intact and PTEN-null primary glioma cell cultures is obtained when using both inhibitors in combination. We next investigated if the effects we observed in culture could be duplicated *in vivo* by treating mice with gliomas for 5 days. The *in vivo* treatments with the combination of CCI-779 and perifosine resulted in decreased Akt and mTOR signaling, which correlated to decreased proliferation and increased cell death independent of PTEN status, as monitored by immunoblot analysis, histology and MRI.

**Conclusions/Significance:**

These findings underline the importance of simultaneously targeting Akt and mTOR to achieve significant down-regulation of the PI3K pathway and support the rationale for testing the perifosine and CCI-779 combination in the human PDGF-subgroup of GBM.

## Introduction

Glioblastoma multiforme (GBM) is both the most common and the most malignant primary brain tumor in adults. Despite aggressive therapy, which includes surgical resection, radiation, and chemotherapy, the survival of GBM patients is poor with median survival of around 1 year [1]. The only recent significant increase in survival for these patients has been obtained by using a combination of radiation therapy with concomitant and adjuvant alkylating chemotherapy (temozolomide), extending mean overall survival by 2.5 months [2]. Many new forms of treatment have been tested, including immunotherapy and gene therapy, but outcomes have not yet been impressive and the development of new treatment modalities is urgent. The PI3K/Akt signaling pathway can be upregulated in gliomas through several mechanisms, most commonly through mutation or loss of heterozygosity of *PTEN* or through amplification/over-expression of critical growth factor receptors such as EGFR and PDGFR. Activation of the PI3K pathway is significantly associated with increased tumor grade, decreased levels of apoptosis, and adverse clinical outcome in human gliomas [3]. Activated PI3K generates phosphatidylinositol 3,4,5-triphosphate (PIP3), which is required for Akt activation [4]. Akt then signals to several downstream targets, including the mammalian target of rapamycin (mTOR). This subsequently leads to increased phosphorylation of eIF4E binding protein 1 (4EBP1) and activation of p70 ribosomal S6 protein kinase (p70^S6K^), which phosphorylates S6 ribosomal protein [5].

Perifosine is alkylphospholipid that interferes with recruitment of Akt to plasma membrane and inhibits Akt phosphorylation and activation. Several mouse and cell culture experiments have demonstrated that perifosine has antitumor activity, which is especially pronounced when combined with radiation [6] or temozolomide [7]. Unfortunately, phase II clinical trials of perifosine as a single agent on recurrent prostate cancer, adenocarcinomas, and melanomas have been disappointing [8], [9], [10].

CCI-779 is a lipid soluble analog of rapamycin that inhibits mTOR by binding to FKBP-12, resulting in cell cycle arrest and decreased growth of several human cancer cell lines [11], [12]. Data from our laboratory using CCI-779 in a mouse model of PDGF-B driven low-grade gliomas demonstrated dramatic anti-proliferative effect in these tumors [13]. In addition to decreased proliferation, there are also many reports of rapamycin promoting pro-apoptotic signals [14], [15], but there is also data supporting its promoting cell survival [16]. Again, data from our lab demonstrated that the blockade of mTOR with CCI-779 resulted in regional apoptosis and conversion in the character of surviving tumor cells from astrocytoma to oligodendroglioma in a mouse model of Akt+KRas-induced GBMs [17].

Data from cell lines and from xenograft experiments indicate the existence of a strong correlation between the anti-proliferative effects of rapamycin analogues and the loss of *phosphatase and tensin homologue gene* (PTEN) [18]. These data lead to the idea that rapamycin and analogs (CCI-779 and RAD001) may be effective in tumors with an activated PI3K-Akt pathway. However, despite pre-clinical data indicating that rapamycin and its analogs have anti-tumor activity, early clinical trials did not show universal anti-tumor activity, especially for tumors with high levels of PI3K-Akt activity, such as glioblastoma [11], [19] and breast cancers [20]. A phase I trial of rapamycin for patients with recurrent PTEN-deficient GBMs demonstrated that rapamycin treatment in around 50% of patients led to Akt activation, which was suggested to be due to the negative feedback [21], [22]. This activation of Akt was associated with shorter time-to-progression during post-surgical maintenance rapamycin therapy [21].

Quantification of growth rates and response to therapy of orthotopic glioma models has been established using MRI [23], [24], [25]. Conventional MRI provides an opportunity to non-invasively follow gross tumor morphology and its evolution over time by exploiting a variety of endogenous tissue properties that allows assessment of gross tumor extent on the resultant MRI contrasts such as T2-weighted and gadolinium-enhanced T1-weighted images. Diffusion-weighted MRI (DW-MRI) can also be used to obtain information related to the microscopic cellular environment of solid tumors [25], [26], [27]. Since water diffusion values are strongly affected by cellular density which impedes water mobility, DW-MRI can be used to characterize highly cellular regions of tumors *versus* acellular regions, to distinguish cystic regions from solid regions, as well as to detect treatment response which is manifested as a change in cellularity within the tumor over time [28]. In this context, we applied DW-MRI to evaluate the effects of molecularly targeted agents using changes in water diffusion values as a surrogate for the underlying changes in tissue structure at the cellular level. MRI provided an opportunity to quantitatively and serially follow therapeutic-induced changes in brain tumor volumes and cellularity.

Based on the results of a phase I human GBM trial [21], we designed a traditional pre-clinical trial using mouse models. Our pre-clinical trial aimed to use the observations made from the human clinical trial [21] and cell line experiments [29], which suggested the possible benefits of using a combination of Akt inhibitors with mTOR inhibitors. Previous studies using various cell lines, including glioma cell lines, have shown enhanced sensitivity of PTEN-deficient tumors to mTOR inhibition [30], which later was demonstrated to be due to Akt dependent regulation of Cyclin D1 and *c-myc* expression [31]. It has been also hypothesized that PTEN status may determine the effectiveness of blocking both the PI3K and mTOR pathways [32]. Other studies utilized PDGF-B driven cortical gliomas to show that perifosine cooperates with temozolomide to arrest cell proliferation *in vivo* [7]. Later, another study using PDGF-B driven Ink4A-ARF null brain stem glioma model demonstrated non-effectiveness of perifosine in a pre-clinical trial as a single agent [33]. In our study we used PDGF-B driven PTEN-deficient and PTEN-intact mouse gliomas generated in *nestin-tv-a/ink4a-arf-/-/pten^fl/^* ^fl^ mice. First, we generated PTEN-deficient and PTEN-intact PDGF-B induced glioma primary cultures (PIGPCs) and treated them with perifosine and CCI-779 both individually and in combination. We showed when these two drugs are combined, Akt and mTOR are uniformly inhibited independent of PTEN status. The benefits of combining perifosine and CCI-779 is further supported with *in vivo* data generated by treating mice with GBMs for 5 days before harvesting tumor tissue for immunoblot and IHC analysis. *In vivo*, perifosine and CCI-779 continued to show synergy in inhibiting the PI3K/mTOR axis corresponding with decreased tumor proliferation and induction of apoptosis. We further monitored the tumor response *in vivo* with MR imaging in PTEN-deficient animals, which demonstrates that the combination induces the greatest loss in tumor cellularity as reflected by changes in ADC value.

## Results

### Generation of PDGF-B induced PTEN-deficient and PTEN-intact high-grade gliomas in Ink4a/ARF null background

**Figure 1:**
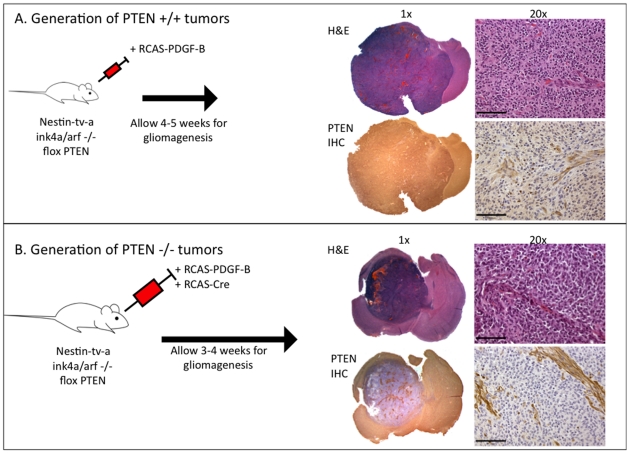
Generation of PTEN wt and PTEN null gliomas using the RCAS/tv-a system. (A) Four-to-six week old *nestin-tv-a/ink4a-arf-/-/pten^fl/fl^* mice are stereotactically injected with DF-1 cells expressing the RCAS-PDGF-B virus and from high-grade gliomas with PTEN intact. (B) Four-to-six week old *nestin-tv-a/ink4a-arf-/-/pten^fl/fl^* mice are stereotactically injected with DF-1 cells expressing the RCAS-PDGF-B virus and DF-1 cells expressing RCAS-Cre virus and from high-grade tumors with PTEN deleted.

All experiments for this study utilized the RCAS/tv-a system to generate PDGF-B driven gliomas in *nestin-t-va/ink4a-arf-/-/pten^fl/fl^* mice [34]. In this background, mice develop tumors with a 90–95% incidence, resulting in the formation of high-grade gliomas within 4–6 weeks post-injection. These tumors share high-grade elements such as microvascular proliferation and pseudopalisading necrosis with human GBMs [34], [35] (Fig. 1A,B) These tumors closely mimic the proneural subtype of GBM, in which *CDKN2A* (encoding for both *p16INK4A* and *p14ARF*) and *PTEN* deletion are observed in up to 56% and 69% proneural human gliomas, respectively [36]. This model also resembles the human pathology in that the tumors generated have abnormal and leaky vasculature, resulting in variable disruption of the blood-brain barrier that can be visualized and quantified by MRI [37]. In order to accurately dissect the role of PTEN in the sensitivity of gliomas to mTOR inhibition and Akt inhibition *in vitro* and *in vivo*, we utilized PDGF-B driven PTEN-deficient and PTEN-intact gliomas. To achieve PTEN deletion in the tumors, we introduced RCAS-Cre in combination with RCAS-PDGF-B to *nestin-t-va/ink4a-arf-/-/pten^fl/fl^* mice, which generated tumors that were PTEN null (Fig. 1B). These mice represent an excellent tool to investigate the role of PTEN status in tumors with otherwise nearly identical genetic backgrounds.

### Perifosine and CCI-779 effectively combine to inhibit the Akt and mTOR pathways in primary glioma cell cultures *in vitro*

**Figure 2:**
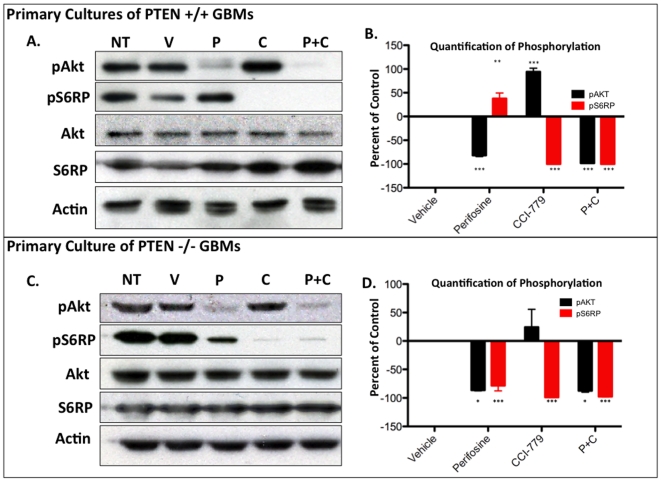
Combing perifosine and CCI-779 effectively inhibits both the Akt and mTOR pathways in primary glioma cultures treated *in vitro*. (A) A representative immunoblot of PTEN +/+ primary GBM cell cultures after 4 hours with no treatment (NT), vehicle (V), 30 µM perifosine (P), 1 nM CCI-779 (C) or combination of 30 µM perifosine and 1 nM CCI-779 (P+C). (B) Quantification of the changes in pAkt and pS6RP based on three independent primary PTEN +/+ GBM cell cultures, with 20 µg protein per well. (C) A representative immunblot of PTEN -/- primary GBM cell cultures after 4 hours with no treatment (NT), vehicle (V), 30 µM perifosine (P), 1 nM CCI-779 (C) or combination of 30 µM perifosine and 1 nM CCI-779 (P+C). (D) Quantification of the change in pAKT and pS6RP based on three independent primary PTEN -/- GBM cultures, with 20 µg protein per well.

In order to determine whether perifosine and CCI-779 were able to effectively inhibit their targeted pathways, we first examined the effects of these inhibitors alone or in combination in PDGF-B induced glioma primary cultures (PIGPCs) derived from the mouse model described above. The cultures used in these experiments were freshly isolated from tumors and passaged a maximum of 5 times. Each PIGPC was generated by combing three freshly isolated tumors, histologically validated to be GBM. First, we performed immunoblots for decrease of pAkt and pS6RP levels with various doses of CCI-779 and perifosine to determine the dose response (data not shown). We chose 30 µM perifosine and 1 nM CCI-779 because treating with those concentrations resulted in reproducible and near complete inhibition of pAkt or pS6RP. We then treated three independently generated PTEN-intact (PTEN +/+) and three independent PTEN-deficient (PTEN −/−) PIGPCs with perifosine and CCI-779 and quantified the changes in pAkt and pS6RP levels to determine the uniformity and statistical significance of the results (Fig. 2 B,D). Untreated PIGPCs demonstrated baseline levels of pAkt and pS6RP, and exposing the cultures to perifosine for four hours resulted in significant decrease in pAkt levels in both PTEN +/+ and PTEN −/− lines by −82.83% (p<0.001) and −86.26% (p<0.05) respectively. However, despite inhibition of pAkt, pS6RP levels remained unaffected in PTEN +/+ glioma cultures. Interestingly, in PTEN −/− glioma cultures, Akt inhibition resulted in incomplete but statistically significant decrease in pS6RP levels −78.33% (p<0.001) (Fig. 2A,B,C,D). These results suggest a differential sensitivity of mTOR in PTEN +/+ and PTEN−/− glioma cultures to Akt inhibition.

After four hours of treatment with 1 nM CCI-779, pS6RP levels were significantly decreased by 100% (p<0.001) and 98.83% (p<0.001) in PTEN +/+ and PTEN −/− cultures respectively. In the case of PTEN +/+ cultures, treatment with CCI-779 resulted in a significant increase in pAkt levels by 94.31% (p<0.001). These results demonstrate the existence of a negative regulatory loop where mTOR inhibition induces the activation of Akt in mouse gliomas, as shown to be the case in various tumor types [22], [38]. For the PTEN −/− cultures the average pAkt increase was 24.46% but failed to reach statistical significance, which can be partially explained with already high levels of pAkt due to PTEN-deficiency. When PICPCs were cultured with a combination of both perifosine and CCI-779, both pAkt and pS6RP levels were simultaneously and significantly decreased. pAkt levels were reduced by 98.18% (p<0.001) and 87.50% (p<.05) and pS6RP levels were reduced by 100% (p<0.001) and 97.70% (p<0.001) in PTEN +/+ and PTEN −/− lines (Fig. 2A,B,C,D).

### PDGF-driven gliomas display lower pAkt and pS6RP levels when glioma-bearing mice are treated *in vivo* with perifosine and CCI-779

**Figure 3:**
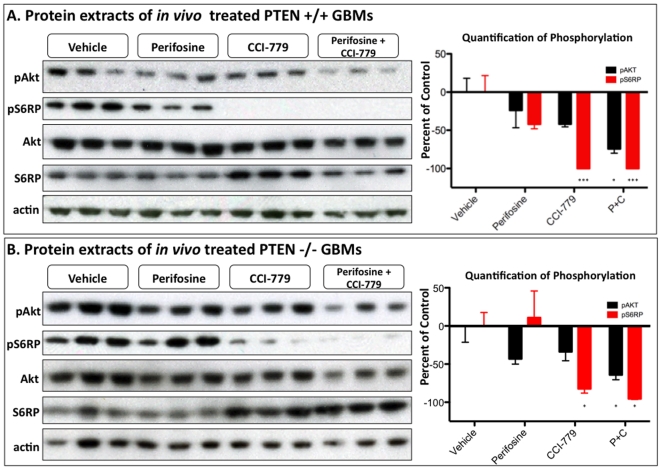
Combing perifosine and CCI-779 effectively decreases the levels of pAKT and pS6RP of gliomas treated *in vivo*. Immunoblot analysis of (A) PTEN +/+ and (B) PTEN -/- tumors after mice with GBMs were treated for 5 days with either vehicle, 30 mg/kg perifosine, 40 mg/kg CCI-779, or a combination of 30 mg/Kg perifosine with 40 mg/Kg CCI-779. The right panel shows the quantification of the changes in pAkt and pS6RP based on comparing the average of each group to the vehicle treated tumors (n = 3 mice per group).

Having established that perifosine and CCI-779 combine to completely inhibit the PI3K/mTOR pathway in glioma cultures, we next wanted to investigate if the two treatments maintained their synergy when used to treat PDGF-B driven gliomas *in vivo*. Twelve PTEN +/+ and twelve PTEN −/− glioma-bearing mice were treated for 5 days by intraperitoneal injection of either vehicle, 30 mg/kg perifosine, 40 mg/kg CCI-779, or a combination of both 30 mg/kg perifosine and 40 mg/kg CCI-779. The animals were sacrificed 24 hours after the last treatment and the tumor tissue was excised and frozen in liquid nitrogen. Once frozen, the tumor was disrupted and lysed to allow for protein collection and immunoblot analysis of changes in pAkt and pS6RP levels (Fig. 3A,B). When both PTEN +/+ and PTEN −/− gliomas were treated with perifosine alone, pAkt levels decreased by 23.79% and 43.13% respectively, although neither decrease reached statistical significance, largely because of the variable pAkt inhibition from tumor to tumor. This is likely due to variability in pAkt levels between individual tumors, as well as the complications of drug delivery caused by the BBB, which may be preventing delivery of an adequate amount of drug to the tumor *in vivo*. We used the highest dose of perifosine tolerated by glioma bearing mice as a single agent or in a combination treatment. Other studies of *in vivo* perifosine activity have reported higher tolerated doses, but those studies utilize subcutaneous tumors as opposed to the intracranial tumors used in this current study [39]. When PTEN +/+ and PTEN −/− tumors were treated with CCI-779, pS6RP was significantly lowered by 100% (p>0.001) and by 82.29% (p>.05), respectively. Interestingly, treating with CCI-779 alone did not increase measured levels of pAKT in either tumor type. When PTEN +/+ and PTEN −/− tumors were treated for 5 days with a combination of perifosine and CCI-779, both pAkt (−74.31% p>.05, −64.17% p<0.05) and pS6RP (−100% p<0.001, −95.45% p<0.05) were significantly decreased. These results demonstrate the enhanced ability of perifosine and CCI-779 to inhibit the PI3K/mTOR pathway when used *in vivo*, and the strongest pathway inhibition occurred when the treatments were used in combination regardless of PTEN status.

### Akt inhibition in combination with mTOR inhibition results in cell death and cell cycle arrest in gliomas

**Figure 4:**
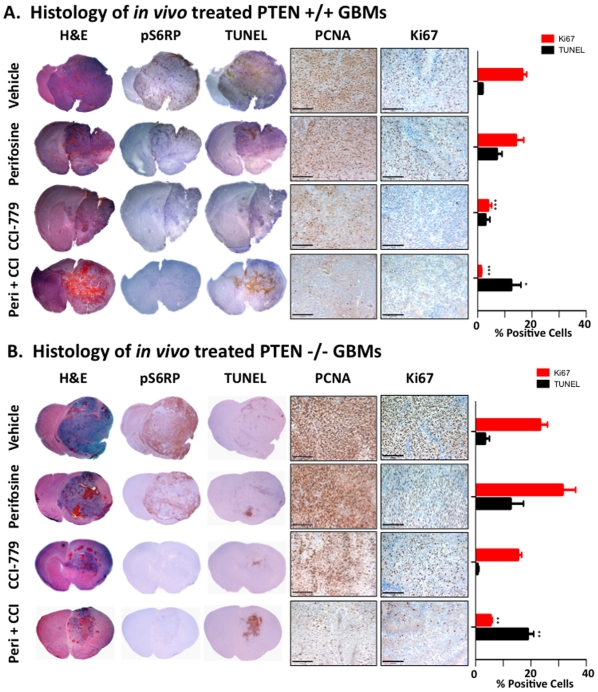
PDGF-B-driven glimoas treated with the combination of perifosine and CCI-779 undergo cell death and have decreased proliferation. Images of H&E, IHC with anti-p-S6RP, anti-PCNA, anti-Ki67, and TUNEL of (A) PTEN +/+ and (B) PTEN -/- GBMs after glioma-bearing mice were treated *in vivo* for 5 days with either vehicle, 30 mg/Kg perifosine, 40 mg/Kg CCI-779, or a combination of 30 mg/Kg perifosine with 40 mg/Kg CCI-779. PCNA and Ki67 images are 200x with the black bar indicating 100 microns. The graph on the right shows quantification of Ki67 and TUNEL staining for 3-4 independent tumors. *,**, and *** represent significance determined by ANOVA analysis for p>0.05, p>0.01, and p>0.001.

We next examined the biological consequences of inhibiting the PI3K/Akt/mTOR axis in PDGF-B driven gliomas. Tumor-bearing mice with PTEN +/+ or PTEN −/− tumors were treated for 5 days as described above (3−4 mice per group), and tumor tissue was analyzed by immunohistochemistry (IHC) for evidence of cell proliferation (anti-PCNA and anti-Ki67) and for cell death (TUNEL). PCNA staining of PTEN +/+ and PTEN −/− gliomas (Fig. 4A,B) demonstrated a slight qualitative decrease in proliferation for perifosine treated animals with a more profound decrease seen in CCI-779 and in the combination treatment groups. The levels of proliferation were further investigated using a second marker for cellular proliferation, Ki67, which had the benefit of less background staining. The Ki67 staining results mirrored those obtained with PCNA, and served as a more accurate marker to use for the quantification presented in the side graph (Fig. 4A,B). Untreated PTEN +/+ tumors had a baseline Ki67 positivity of 16.52 +/− 3.12%, which was unchanged by perifosine (14.14 +/− 5.74%), but dramatically decreased by treatment with CCI-779 and combination therapy (3.86 +/− 2.65% and 1.22 +/− 0.61%, p>0.001). The decreased proliferation with CCI-779 coincides with a downregulation of pS6RP. PTEN −/− tumors had a higher Ki67 positivity (23.36 +/− 5.28%) that was not significantly changed by perifosine (31.47 +/− 9.45% p = 0.18). Treatment with CCI-779 again resulted in decreased levels of pS6RP, and Ki67 positivity was decreased to 15.38 +/− 2.67%, though this was not statistically significant. Interestingly, the decrease in proliferation with CCI-779 alone was less dramatic in the PTEN −/− tumors than in PTEN +/+ tumors, as can be seen by both the PCNA and the Ki67 staining. When both drugs were combined to treat PTEN −/− gliomas, Ki67 positivity was significantly decreased to 5.46% +/− 1.29% (Fig. 4A,B).

Finally, TUNEL staining for the presence of apoptosis revealed increased levels of cell death in tumors treated with perifosine in both PTEN +/+ (5.25% increase) and PTEN −/− tumors (9.31% increase), though the increases were not statistically significant due to varying degrees of apoptosis from tumor to tumor (Fig. 4A,B). This variability mirrors the variability in Akt inhibition seen in the immunoblot analysis (Fig. 3A,B), again suggesting that the variability in the drug's delivery may lead to differing effectiveness from tumor to tumor. CCI-779 treated mice did not demonstrate increased TUNEL positivity. In both tumor types, the combination treatment group displayed statistically significant increase in TUNEL positivity (PTEN +/+ = 10.60% increase, PTEN −/− = 15.48% increase) (Fig. 4A,B). Independent of PTEN status, the combination of perifosine and CCI-779 inhibited pS6RP signaling while inducing a qualitative and quantitative decrease in proliferation and increase in cell death.

### Imaging characteristics of gliomas treated with perifosine, CCI-779 and combination in PTEN-deficient animals

**Figure 5:**
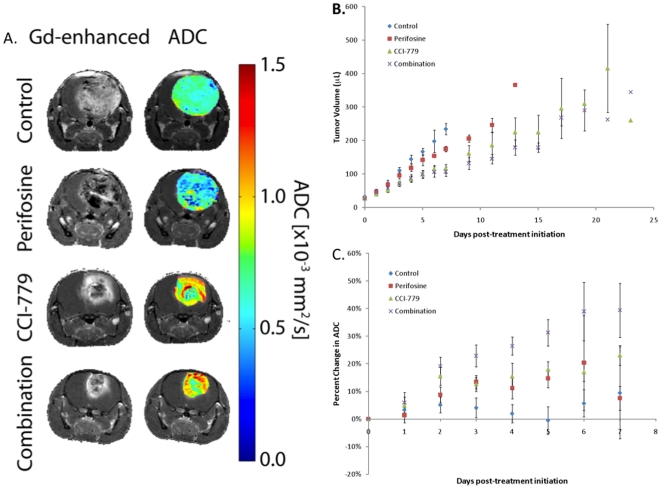
*In vivo* imaging of therapeutic response using contrast enhanced diffusion weighted MRI. (A) MR gadolinium (Gd) contrast-enhanced T1-weighted images and ADC color overlay maps demonstrate effects of *in vivo* effects of treatment with obtained from representative control, perifosine, CCI-779 and combination (perifosine + CCI-779) treated glioma mice at 5-7 days post-initiation of therapy. (B) Plot of mean MRI-determined tumor volumes based upon Gd-contrast enhanced regions versus time post-treatment initiation for control, perifosine, CCI-779 and combination (perifosine + CCI-779) treated mice. (Error bars ± SEM). (C) Plot of percent change of mean ADC values versus time post-treatment initiation for control, perifosine, CCI-779 and combination (perifosine + CCI-779) treated mice. (Error bars ± SEM).

Imaging studies were accomplished using the PTEN-deficient gliomas model to evaluate the effects of P13K/mTOR pathway inhibition on tumor growth rates and cellularity. In order to characterize the *in vivo* tumor response, we followed tumors with MR imaging during the treatment window. MR images of animals in the four treatment groups were obtained daily during treatment in order to follow both tumor volume and treatment-associated alterations in tumor water diffusion values. Shown in Figure 5A are representative contrast-enhancing MR images and the same MR images with color overlay ADC maps of the tumors for each of the treatment groups shown for 24 hours after the last treatment dosage. Tumor volumes were obtained from the contrast-enhanced images and plotted over time as shown in Figure 5B. At day 3 post-treatment initiation, animals treated with CCI-779 either alone or in combination had generated a statistically smaller tumor volume than controls (p<.05), which continued throughout the study. By day 7, the perifosine treated cohort was found to have a significantly larger tumor volume than both the CCI-779 and combination treatment cohorts. In contrast, perifosine treated tumor volumes were not found to be different from controls at any point during the study. The same was true between the CCI-779 treated groups.

DW-MRI was used to assess changes in tumor cellularity during treatment. Quantitative DW-MRI maps are shown as color overlays on their corresponding anatomical images in Figure 5A. At day 2 post-treatment initiation, combination therapy had a significant increase in ADC values from control values (p<.05). By day 4, the combination therapy generated a significantly larger percent change in ADC than perifosine treated animals. CCI-779 reached significantly higher ADC values versus control on day 5 post-initiation of treatment (Fig. 5C). No additional group differences in the percent change in ADC values between groups were found to be significant. Nevertheless, changes in ADC values between combination and CCI-779 therapies had resulted in a p = 0.1 by day 5 post-treatment initiation.

## Discussion

Our studies using primary glioma cultures from PTEN-deficient and PTEN-intact PDGF-B driven mouse GBMs demonstrate that PTEN status influences mTOR's sensitivity to Akt inhibition with perifosine. When PTEN is intact, mTOR appears to be AKT independent. Our data agrees with recent studies in EGFR driven gliomas models that show mTOR can be regulated by the PKC pathway independent of Akt phosphorylation [40]. Gliomas are one of the many tumor types where selectively inhibiting key signaling pathways appears to be a moving target. Recent studies have shown that in response to therapy, gliomas undergo critical changes in their molecular circuitry, such as loss of key tumor suppressor proteins, the selection for kinase-resistant mutants, and the deregulation of feedback loops [41]. Our model supports the view that the presence or absence of a single regulatory component, such as PTEN, is enough to shift the signaling network, even in very similar tumors. However, tumors treated with a combination of perifosine and CCI-779 had dramatically decreased the levels of pAkt and pS6RP independent of PTEN status.

Synergistic effects between rapamycin and LY294002, an PI3K inhibitor that acts upstream of Akt, are commonly observed *in vitro* [29], [42], but confirming those results in clinically relevant *in vivo* models has proved to be more difficult. Since perifosine was able to penetrate the BBB in previous mouse glioma studies [7], [33], we were able to follow up on the effective synergy we observed in our *in vitro* culture studies and investigate the effectiveness of this combination in PTEN-intact and PTEN-deficient PDGF-B-driven Ink4a-ARF-deficient gliomas *in vivo*. Five days of *in vivo* treatment with perifosine resulted in variable but not statistically significant decreases of pAkt levels. Similarly, perifosine alone caused a variable and not statistically significant increase cell death. Since in cell culture, perifosine decreased pAkt levels dramatically and in a dose-dependant manner (data not shown), a possible explanation of the lack of statistically significance *in vivo* could be as a result of low drug concentrations in the tumor due to the BBB. To further investigate this possibility, we treated tumor-bearing mice with a single dose of 120 mg/kg perifosine, and immunoblot analysis showed a statistically significant decrease in pAkt levels at 3 and 6 hours (data not shown). This further supports the hypothesis that, in our model, perifosine levels are limited by the BBB, and that we might be able to overcome this complication with an increased dose of perifosine. However, these elevated doses of perifosine were not well tolerated by the animals.

It is also worth addressing that, unlike the results observed in culture, Akt inhibition caused by perifosine alone did not have a drastically different effect when comparing the PTEN +/+ and PTEN −/− tumors. One possible explanation for this discrepancy is that the degree of Akt inhibition *in vivo* was much less dramatic than that observed in culture. When performing experiments in culture, we have precise control over both the concentration of the drug and the length of exposure to the drug. These parameters cannot be fully reproduced *in vivo*. While the exact cause for the discrepancy between these findings is a potential area for future study, it was encouraging that significant Akt inhibition was achieved when combined with CCI-779, further supporting the rational for using the combination of these inhibitors.

In contrast to perifosine, CCI-779 was much more consistent as a single agent, suggesting the BBB was not a complicating factor for this compound. This correlates with human clinical data, were CCI-779 has been shown to effectively penetrate the BBB of patients with recurrent glioblastoma [43]. Treating animals for 5 days with 40 mg/kg decreased pS6RP levels in tumors independent from PTEN status, and the decrease in pS6RP corresponded with a decreased cellular proliferation rate. Of note, this effect was more dramatic in PTEN intact tumors, as demonstrated by both PCNA and Ki67 staining. While gliomas treated with CCI-779 *in vivo* did show decreased pS6RP, they did not show increased pAkt levels. Interestingly, our results did not completely reproduce data observed by Cloughsey *et al* [21] where fourteen recurrent PTEN null GBMs were biopsied both before and after treatment with rapamycin, in which half of the patients had no change in Akt activity and half had a slight increase in Akt activity. Although the human GBMs were selected based on PTEN status, they were not selected based on their molecular subgroup status. Recent studies have clearly shown at least three distinct subtypes of GBM, and these subtypes have very different molecular and signaling profiles[36], [44]. By contrast, our study specifically focuses on PDGF-driven gliomas in the hope to better understand how the human PDGF-subgroup will respond to therapy. However, we do not believe we can compare our results in modeling PDGF-driven glioma to all subtypes of glioma, and thus we cannot compare our results directly to the previous human clinical trials.

When the two drugs were combined *in vivo* for 5 days, both pAkt and pS6RP levels were significantly decreased independent of PTEN status. This coincided with a striking decrease in cellular proliferation and a marked increase in cell death, although the relative decrease in proliferation and increase in cell death was greatest in PTEN null tumors. Therefore, we utilized PTEN-deficient tumors and MR-imaging to monitor the dynamics of the tumor response in real time by monitoring *in vivo* changes in cellularity and apparent diffusion value (ADC) changes and imaging characteristics. MRI allowed for tumor staging and thus gliomas could be entered into the study at a similar size, providing an opportunity to follow changes in tumor volumes over time for each of the four animal cohorts. In Figure 5, the quantified diffusion values are provided as ADC values and were overlayed on the corresponding anatomical image in order to visualize the spatial variations of the changes within the tumor mass. If tumor cell killing occurs during treatment intervention, ADC values will increase in those regions of the tumor affected. What Figure 5 specifically shows is that the ADC values progressively increased from controls to Perifosine to CCI to combination therapy, as visualized by the increased intensity represented by the red regions in the image. As the ADC values are a reflection of the rate of cellular removal, these results indicate a progressive loss of cell viability over these 4 cohorts of animals.

It is interesting to note that tumor volume continued to gain during the treatment period, despite the marked increase in diffusion values. Since the gliomas had a rapid proliferation rate, it may be possible that the rate of cell removal, as evidenced by the increased ADC values (red regions in the color images), never quite overcame the rate of cellular proliferation, even in the combination treatment group. In the current study, changes in ADC values appear to correlate well with the reduction of tumor growth rates and serve as an indication of the efficacy of the treatment. Although overall tumor volume did not shrink, ADC values proved to be an exquisitely sensitive measurement for comparing different treatments, and this imaging biomarker can be easily implemented in clinical translation of promising therapies. We would anticipate that future treatments capable of producing tumor mass shrinkage would be reflected as an even greater response in tumor ADC values, and that this strategy would serve as a foundation on which to non-invasively monitor novel treatment protocols. Thus, DW-MRI may provide an opportunity to assess early treatment response in patients treated with agents targeting the PI3K/Akt/mTOR pathways.

In conclusion, Our preclinical data strongly supports the notion that the best clinical potential for inhibiting the PI3K/Akt/mTOR will be reached when multiple components of the pathway are inhibited and that DW-MRI may be used as an early imaging response biomarker to provide quantitative and spatial information related to tumor cell death during treatment administration. Furthermore, our studies represent the murine portion of a co-clinical trial where the human part of the trial is currently being run at Memorial Sloan Kettering Cancer Center [NCT01051557]. The trial is recruiting adults with recurrent gliomas to be treated with CCI-779 and perifosine as salvage therapy. Therefore, further insight as to how these two inhibitors work together, specifically in a PDGF-driven glioma model, is of significant importance to understanding the potential impact on the human disease.

## Materials and Methods

### Ethics Statement

All of the animal experiments were conducted using protocols approved by the Institutional Animal Care and Use Committees of Memorial Sloan-Kettering Cancer Center and the University of Michigan Medical School. The approved protocols are 00-11–189 (MSKCC, last approved 3/15/2010) and 09583 (UMMS, last approved 11/25/09).

### Cell culture and transfection

Df-1 cells were purchased from ATCC. Cells were grown at 39°C according to ATCC instructions. Transfections with RCAS-PDGF-B-HA or RCAS-Cre were performed using Fugene 6 transfection kit (Roche # 11814443001) according to manufactures instructions.

### Generation of tumors using RCAS/tv-a

4–6 week-old *nestin-tv-a/ink4a-arf-/-/pten^fl/fl^* mice were anesthetized with ketamine (0.1 mg/g) and xylazine (0.02 mg/g) and injected using stereotactic fixation device (Stoelting, Wood Dale, IL). One microliter of RCAS-PDGF-B or 1∶1 mixture of 4 ×10^4^ RCAS-PDGF-B and RCAS-Cre transfected DF1 cells was delivered using a 30-gauge needle attached to a Hamilton syringe. Cells were injected to the right frontal cortex, coordinates bregma 1.7 mm, Lat −0.5 mm, and a depth 1.5 mm. Mice were monitored carefully and sacrificed when they displayed symptoms of tumor development (lethargy, head tilt).

### Generation of PDGF-B driven primary gliomas cultures

Tumors were dissected and enzymatically digested in Hanks balanced salt solution containing 12% papain and 10 µg/ml DNase at 37°C for 15 min, with subsequent inactivation using ovomucoid (1 mg/mL) (Worthington, Lakewood, NJ, USA). The cell suspension was consecutively washed and resuspended to obtain a single cell suspension, which was resuspended in DMEM media containing 10% FBS, 2 mM L-glutamine, 100 units/mL penicillin, and 100 µg/mL streptomycin.

### Drug administration

Tumor-bearing mice received intraperitoneal injection of 30 mg/kg Perifosine (Keryx Biopharmaceuticals, NY) in 0.9% NaCl daily for 5 days. CCI-779 (Wyeth, Pearl River, NY) was given through intraperitoneal injection daily at the dose of 40 mg/kg for 5 days. The drug was first dissolved to 50 mg/ml in 100% ethanol and then diluted to 2 mg/ml with 5% Tween-80 (Sigma) and 5% polyethylene glycol-400 (Sigma). For combination treatment, mice received 30 mg/kg perifosine and 40 mg/kg CCI-779 simultaneously for 5 days.

### Tissue Processing

Animals used for histological analysis and immunoblot analysis were sacrificed 24 hours after the last dose of drug treatment and transcardially perfused with 4°C PBS. At this point, tumors for western analysis were excised and frozen in liquid nitrogen and tumors for histological analysis were further perfused with 10% neutral buffered formalin before being excised and fixed for 72 hours. Fixed tissues were then embedded in paraffin. Formalin-fixed, paraffin embedded specimens were serially sectioned, 5 µm slides were mounted.

### Immunoblot Analysis

For cell culture analysis, low passage PIGPCs (p≤5) were plated in DMEM media with 1% FBS and treated with either DMSO+PBS, perifosine, CCI-779, or combination. After 4 hours, the cells were rinsed twice with PBS and scraped into lysis buffer (1% NP-40, 0.1% Triton X-100, 150 mM NaCl, 50 mM Tris pH 7.5, 1 mM EDTA, 1 mM PMSF, 1 mM Na_3_VO_4_, 250 mM NaF). For *in vivo* tumor analysis, fresh frozen tumors were manually disrupted with a mortar and pestle and resuspended in lysis buffer. For each sample, 20 µg of lysate was loaded for SDS-PAGE and transferred to a PVDF membrane. The antibodies used included anti-phospho-Akt (Ser^473^) (Cell Signaling #4060), anti-phospho-S6RP (Ser^235/236^) (Cell Signaling #4856), anti-Akt (Cell Signaling #4685), anti-S6RP (Cell Signaling #2217), anti-actin (Chemicon MAB1501). Immunoblots were quantified using ImageJ software [45].

### Immunohistochemistry

For immunohistochemical detection, an automated staining processor was used (Discovery, Ventana Medical Systems, Inc.). The protocols were established at the Molecular Cytology Core Facility and Eric Holland laboratory at MSKCC. Primary antibodies used were anti-pS6RP 1∶300 (Cell Signaling, # 2211), anti-PCNA 1∶2000 (DAKO Cat# M0879), anti-PTEN 1∶100 (Cell signaling, #9559), and anti-Ki67 1∶200 (Dako Cat.# M7240). Ki67 quantification was performed using HistoFACS and HistoQUEST and HistoFACS software (TissueGnostics), and four tumors were analyzed for each group.

### TUNEL assay

The terminal deoxynucleotidyl transferase-mediated dUTP-biotin nick-end labeling (TUNEL) assay was performed on 5 µm sections by using Terminal Transferase, recombinant kit (Roche, #3333566) in accordance with the manufacturer's protocol. TUNEL quantification was performed HistoFACS and HistoQUEST and HistoFACS software (TissueGnostics) and four tumors were analyzed for each group.

### MRI Image Processing and Analysis

MRI scans were performed on a 9.4T, 16 cm horizontal bore Varian, Inc (Palo Alto, CA) *Direct Drive* system with a SAW mouse head coil (m2m Imaging, Corp., Cleveland, OH). During all MRI procedures, the animals were anesthetized with 1–2% isoflurane/air mixture, and body temperature was maintained using a heated air system (Air-Therm Heater, World Precision Instruments, Sarasota, FL). MR images were acquired prior to treatment initiation, daily for a week and every other day thereafter till the animal expired or became moribund and was then sacrificed. Each MRI experiment per animal consisted of a contrast-enhanced T1-weighted spin-echo sequence, used for delineating tumor from healthy brain tissue, and a diffusion weighted MRI sequence.

Contrast-enhanced T1-weighted spin-echo images were acquired with the following parameters: Repetition time/echo time (TR/TE) = 85/3.2 ms, field of view (FOV) = 30 mm, matrix size  = 128×64, slice thickness  = 1 mm, 13 slices and number of scans  = 2. Contrast-enhancement was performed by i.p. administration of 50 µl of 0.5 M gadolinium-DTPA (Magnevist, Bayer Healthcare Pharmaceuticals, Wayne, N.J) 2 minutes prior to image data acquisition.

Diffusion-weighted images were acquired using a spin-echo sequence, with motion correction using a navigator echo and gradient waveforms sensitive to isotropic diffusion, with the following parameters: TR/TE  = 4000/47 ms, FOV  = 30 mm, matrix size  = 128×64, slice thickness  = 1 mm and b-values (diffusion weighting) of 120 and 1200 s/mm^2^.

Volumes of interest (VOIs) were contoured around the enhancing rim of the tumors on the contrast-enhanced T1-weighted images for volume measurements and determination of whole-tumor means of ADC. ADC was calculated from two diffusion weightings (b-values) as previously described [26]. Diffusion measurements were calculated as means over the tumor volume. All image reconstruction and digital image analysis was accomplished using in-house programs developed in the software application Matlab (The Mathworks, Natick, MA, USA).

### Statistics

Graphs have been made using GraphPad Prism 4 (GraphPad Software, San Diego, CA) and analyzed using ANOVA. Figure 1C,F; Figure 2A,B; Figure 3A,B were analyzed used Dunnett's Multiple Comparison Test of ANOVA, which compares all columns versus control column. Statistical analysis of the MRI analysis utilized ANOVA with a Bonferroni *post hoc* test to account for multiple comparisons. Group comparisons of percent change in tumor volume and ADC were assessed at individual time points using an independent sample Student's *t*-test. All statistical computations were performed with a statistical software package (SPSS Software Products, Chicago, IL). * = p<0.05, ** = p<0.01, *** = p<0.001, absence of star = not significant.
