# Correction: Perifosine and CCI 779 Co-Operate to Induce Cell Death and Decrease Proliferation in PTEN-Intact and PTEN-Deficient PDGF-Driven Murine Glioblastoma

**DOI:** 10.1371/annotation/66641ad6-afb9-4d3c-ade6-73fcd5aab061

**Published:** 2011-01-27

**Authors:** Kenneth L. Pitter, Craig J. Galbán, Stefanie Galbán, Omid Saeed Tehrani, Fei Li, Nikki Charles, Michelle S. Bradbury, Oren J. Becher, Thomas L. Chenevert, Alnawaz Rehemtulla, Brian D. Ross, Eric C. Holland, Dolores Hambardzumyan

The fourth author's name is misspelled. The correct spelling is: Omid Saeed Tehrani. The correct citation is: Pitter KL, Galbán CJ, Galbán S, Tehrani OS, Li F, et al. (2011) Perifosine and CCI 779 Co-Operate to Induce Cell Death and Decrease Proliferation in PTEN-Intact and PTEN-Deficient PDGF-Driven Murine Glioblastoma. PLoS ONE 6(1): e14545. doi:10.1371/journal.pone.0014545

